# In Vitro Synergistic Antibacterial Effects of Extract and Honey Derived from Nigella Sativa on Streptococcus Mutans: Antibacterial Effects of Nigella Sativa Extract and Honey

**DOI:** 10.31661/gmj.v13iSP1.3567

**Published:** 2024-12-08

**Authors:** Seyed-Ali Kamali, Mohammad-Bagher Rezvani, Maryam Pourhajibagher, Fatemeh Farzaneh, Hanie-Sadat Emami-Razavi

**Affiliations:** ^1^ Department of Operative Dentistry, Faculty of Dentistry, Shahed University, Tehran, Iran; ^2^ Dental Research Center, Dentistry Research Institute, Tehran University of Medical Sciences, Tehran, Iran

**Keywords:** Nigella Sativa, Streptococcus Mutans, Honey, Herbal Medicine

## Abstract

**Background:**

This study aimed to assess the synergistic antibacterial effects of Nigella sativa extract and honey on Streptococcus mutans.

**Materials and Methods:**

In this in vitro study, the minimum inhibitory concentrations (MICs) and minimum bactericidal concentrations (MBCs) of extract and honey derived from N.sativa were determined. The antibacterial activity of 1%, 2%, 3%, 4% and 5% extract and honey derived from N.Sativa was then evaluated against S. mutans by the agar disc diffusion method. Data were analyzed by ANOVA followed by pairwise comparisons by the Tukey’s test and paired sample t-test.

**Results:**

N. sativa extract showed inhibitory effects on S. mutans. The MIC was 250 mg/mL for 1% and 2% concentrations and 125 mg/mL for 3%, 4%, and 5% concentrations of the mixture against S. mutans. In addition, the MBC was determined to be 500 mg/mL for the 1% and 2% concentrations, while it was 250 mg/mL for the 3%, 4%, and 5% concentrations. However, N. sativa honey did not have any antibacterial activity against S. mutans. Combination of N. sativa extract and honey had a significant inhibitory effect on S. mutans. Amount of 1000 mg/mL concentration of 5% honey and N. sativa caused the largest growth inhibition zone (13 mm) with no significant difference with 0.2% chlorhexidine as control (12 mm) (P=0.74).

**Conclusion:**

N. sativa honey alone did not demonstrate any significant effect on inhibiting the growth of S. mutans. The combination of N. sativa and honey had an inhibitory effect on the growth of S. mutans. Increased concentration of N. sativa result in a greater inhibition of S. mutans growth.

## Introduction

Dental caries is the most common non-communicable disease, affecting 80% to 90% of the world’s population. According to the World Health Organization, approximately half of the world’s population (approximately 3.6 billion people) had dental caries in 2016 [1][2]. Dental caries is a costly disease, accounting for 5% to 10% of the healthcare budget of industrial countries [3].

Acidogenic and aciduric bacteria such as Streptococcus mutans (S. mutans), and Lactobacillus are responsible for caries development. S. mutans has the most prominent role in this regardand it can be abundantly isolated from carious lesions of humans and animals consuming high-carbohydrate foods [3]. Foods rich in carbohydrates and acidogenic strains of S. mutans were long regarded as the main etiology of dental caries. However, recent investigations on S. mutans DNA and RNA revealed that it is capable of creating a biofilm and resist the effects of antibiotics [2][4].

Since S. mutans is the main cause of dental caries, the majority of caries control strategies have focused on this microorganism [5][6].

Some other bacteria may also play a role in development of caries such as Leptotrichia, Rothia, and Veillonella that are involved in the occurrence of enamel caries, and Atopobium, Pseudoramibacter, Schlegelella, Lactobacillus and Streptococcus sanguinis that are involved in development of dentin caries [7]. Inhibition of S. mutans can inhibit caries progression [8][9].

Nigella sativa (N. sativa) from the family of Ranunculaceae is a miracle herb with a long history in traditional medicine. It is native to Southern Europe, North Africa, and south-west Asia, and is cultivated in many Mediterranean and Middle Eastern countries [10]. It has a wide range of therapeutic effects with diuretic, anti-hypertension, anti-diabetic, anti-cancer, immunomodulatory, analgesic, antimicrobial, anti-parasite, anti-inflammation, anti-spasmolytic, bronchodilator, and antioxidant properties [11][12].

Evidence shows that N. sativa has inhibitory effects on S. mutans and can prevent caries as such [13][14].

Commercial synthetic mouthwashes such as chlorhexidine and Listerine are commonly used to decrease the oral microbial load [15][16].

However, due to fewer side effects and higher cost-effectiveness of herbal extracts, researchers have focused on their possible incorporation in the formulation of mouthwashes, given that their safety and optimal antibacterial activity against cariogenic microorganisms are confirmed. N. sativa extract has four main alkaloid constituents. It contains thymoquinone, thymol, thymohydroquinone, and di-thymoquinone as effective substances of its aqueous extract [17][18][19]. Also, P-cymene is a major constituent of its extract, which accounts for higher weight percentage of its volatile oil as well [19]. N. sativa contains lipids, vitamins, minerals, proteins (8 essential amino-acids) and carbohydrates [20][21].

It is also a rich source of essential and non-essential fatty acids. Its main unsaturated fatty acids include linoleic and oleic acids. It also contains phospholipids, carotene, calcium, iron, and potassium [22][23]. It has antioxidant, anti-inflammatory, anti-histaminic, and immunosupportive effects as well. It can lower the blood glucose and lipid levels, blood pressure, and uric acid [24].

The antibiotics that are effective against S. mutans such as ampicillin, cephazolin, methicillin, and clindamycin have side effects such as nausea and vomiting, metallic taste, joint pain, deglutition pain, and stomach burning sensation [25][26]. Thus, alcoholic extract of N. sativa has been considered as an alternative to antibiotics due to its confirmed antibacterial effects [27][28].

Honey, despite its sweetness, has an inhibitory effect on a number of Gram-positive and Gram-negative bacteria and particularly S. mutans [15][16]. However, its antibacterial activity varies depending on its location of harvesting, type of bees, and honey composition, which may vary from one geographical region to another [18].

Although numerous studies have evaluated the antibacterial activity of N. sativa and honey on S. mutans, their synergistic antibacterial effect has not been previously investigated. Thus, this study aimed to assess the synergistic antibacterial effects of N. sativa extract and N. sativa honey on S. mutans.

## Materials and Methods

This in vitro, experimental study was approved by the ethics committee of the School of Dentistry of Shahed University (IR.SHAHED.REC.1400.192)

### Obtaining the Extract and Honey

N.sativa seeds was obtained from the Traditional Medicine Department of Tehran University of Medical Sciences. Its ethanolic extract was subsequently obtained in 70% concentration (200 g of N. sativa in 1000 mL of ethanol). [13] After 72 hours, the solution was filtered through a #1 Whatman filter paper with 150 µm pores, and its ethanol was evaporated by using a water bath (Gesellschaft fu ¨r LabortechnikGmbH, Burgwedel, Germany). The extract was then stored at 4°C. Since the extract was not water soluble, dimethyl sulfoxide was added to it. The N. sativa honey was also collected for this study from the bees fed from N. sativa flower of Iran’s central area (Isfahan). To mix the N. sativa extract and honey, 5 different weight percentages of the N. sativa extract (1% to 5% w/w), and 95-99% w/w raw honey were used (for instance, 3% N. sativa plus 97% honey). To prepare different concentrations of the mixture, it was serially diluted (from ½ to 1/128 v/v), then 8 different concentrations for each mixture was provided.

### S. Mutans Culture

Standard-strain S. mutans (ATCC35668) was purchased from the Iranian Biological Resource Center. Then were cultured in 5 ml brain heart infusion (BHI) broth (Merck, Germany) at 37°C in the presence of 5% CO2 [29]. After that a suspension with a concentration of 1.5 × 108 CFU/mL (equal to 0.5 McFarland) was prepared from it.

### Determination of Minimum Inhibitory Concentration (MIC)

The MIC values were determined according to the Clinical and Laboratory Standards Institute (CLSI) standard [30]. In brief, 100 µL of BHI broth was added to the wells of a 96-well microplate. Next, 100 µL of each mixture was added to the first well in final concentration of 1 g/mL. Subsequently, 100 µL of the contents of the first well was transferred to the second well. This process was continued until the 10th well, and finally, 100 µL of the contents of the last well was discarded. Next, 100 µL of bacterial suspension containing 1.5 × 106 CFUs/mL was added to each well, and the microplate was incubated at 37°C and 5% CO2 foe 24 hours.

Wells containing 200 µL of BHI broth served as the negative control while wells containing BHI broth and bacteria served as the positive control. The concentration of mixture in the first well that inhibited visible bacterial growth (turbidity) was recorded as the MIC.

### Determination of Minimum Bactericidal Concentration (MBC)

According to the CLSI guideline [31] MBC is identified as the lowest concentration of an antimicrobial agent that can decrease the bacterial population by 99.9% after 24 hours. To determine the MBC, after identifying the MIC, the microplate wells containing the MIC and one higher concentration were inoculated onto BHI agar medium (Merck, Germany). The plates were then incubated at 37°C in an environment with 5% CO2 for 24 hours. The concentration of mixture at which no bacterial growth was observed was designated as the MBC.

### Assessment of Antibacterial Activity by the Agar Disc Diffusion Technique

Paper discs with 6 mm diameter were dipped in 20 µL of the N. sativa and honey mixture in different concentrations (similar to those used for MIC determination). S. mutans suspension with 0.5 McFarland standard concentration was prepared in BHI broth and swab-cultured on BHI agar (Merck, Germany). The discs were placed on the plate surface with 2 cm distance from each other. The plates were then incubated at 37°C and 5% CO2 for 24 hours. The diameter of the growth inhibition zones was finally measured by a ruler and reported in millimeters. Chlorhexidine at a concentration of 0.2% was used as the positive control group.

### Statistical Analysis

All tests were conducted three times.Normal distribution of data was ensured by the Kolmogorov-Smirnov test. Thus, data were analyzed by ANOVA followed by pairwise comparisons by the Tukey’s test. Differences in growth inhibition zone diameters were analyzed by paired sample t-test. P<0.05 was considered statistically significant.

## Results

**Table:** Table1. MIC of the Mixture of N. sativa Extract and Honey in Different Concentrations Against S. mutans

**Concentration**	**MIC (mg/mL)**	**MBC (mg/mL)**
1%	250	500
2%	250	500
3%	125	250
4%	125	250
5%	125	250

### MIC and MBC

Table-1 shows the MIC and MBC of the mixture of N. sativa and honey in different concentrations against S. mutans. As shown, by an increase in concentration, the antimicrobial activity of the mixture increased. As shown, the MIC was 125 mg/mL for 3%, 4%, and 5% concentrations of the mixture. While it was 250 mg/mL for 1% and 2% concentrations. Additionally, the MBC was found to be 500 mg/mL for the 1% and 2% concentrations, whereas the MBC for the 3%, 4%, and 5% concentrations was 250 mg/mL.

### Agar Disc Diffusion Results

No growth inhibition zone was seen around the discs containing different concentrations of 1% honey and N. sativa. However, 1000 mg/mL concentration of 2% and 3% honey and N. sativa created a S. mutans growth inhibition zone by 9 mm. Also, 500 and 1000 mg/mL concentrations of 4% honey and N. sativa caused growth inhibition zones with 10 and 8 mm diameter, respectively. Moreover, 500 mg/mL concentration of 5% honey and N. sativa created a 10 mm growth inhibition zone (Table-2);1000 mg/mL concentration of 5% honey and N. sativa caused the largest growth inhibition zone (13 mm); however, it had no significant difference with 0.2% chlorhexidine as control (12 mm) (P=0.74).

## Discussion

**Table:** Table2. Diameter of S. Mutans Growth Inhibition Zones Caused by Different Concentrations of Honey and N. Sativa Extract Mixture

**Concentration of mixture**	**Growth inhibition zone diameter (mm)**
1%	-
2% of first concentration	9
3% of first concentration	9
4% of first concentration	10
4% of second concentration	8
5% of first concentration	13
5% of second concentration	10
Negative control (honey)	-
Positive control (chlorhexidine 0.2%)	12

This study assessed the synergistic antibacterial effects of N. sativa extract and honey on S. mutans. Since thymoquinone, which is the effective substance of N. sativa, has toxic effects in high concentrations [31], low percentages of N. sativa were used in the present study. Also, honey was added to it to improve its taste. The results showed that N. sativa extract had inhibitory effects on S. mutans; however, N. sativa honey did not show any antibacterial activity. Combination of N. sativa extract and honey had inhibitory effects on S. mutans. Chaieb et al. [32] evaluated the antibacterial activity of thymoquinone, and reported that it had greater effects on Gram-positive bacteria. It also impaired bacterial adhesion. Gawron et al. [33] assessed the effect of N. sativa extract on methicillin-resistant Staphylococcus aureus, and showed that its antimicrobial activity depended on the concentration of thymoquinone, and could have significant bacteriostatic and bactericidal effects. Mouwakeh et al. [34] evaluated the effect of N. sativaextract and bioactive materials on Staphylococcus aureus and confirmed its optimal antibacterial activity. The above-mentioned two studies evaluated Staphylococcus aureus, which is a Gram-positive facultative anaerobe similar to S. mutans. Consistent with their results, the present study showed concentration-dependent antibacterial activity of N. sativa extract against S. mutans.

The present results revealed the inhibitory effect of the mixture of N. sativa extract and honey on growth of S. mutans in agar disc diffusion technique. Similarly, previous studies showed significant antibacterial activity of N. sativa extract against antibiotic-resistant bacterial species [33][34]. Dalli et al. [35] evaluated the molecular composition and antimicrobial activity of N. sativa on drug-resistant bacteria. They evaluated N. sativa from different geographical sources and showed their variable antimicrobial activity. For instance, N. sativa collected from India had the highest inhibitory effect on Escherichia coli followed by N. sativa collected from Saudi Arabia, Morocco and Syria. However, determination of their MIC revealed that all N. sativa types had an inhibitory effect on Escherichia coli in concentrations equal or higher than 20 mL. They also showed the optimal antibacterial effect of N. sativa on Pseudomonas aeruginosa and Acinetobacterbaumannii[35]. Rostinawat et al. [13] showed the anti-S. mutans activity of the ethanolic extract of N. sativa in 400 to 1200 mg/mL concentrations. The diameter of the growth inhibition zone increased by an increase in concentration of N. sativa. Assessment of MIC revealed no bacterial growth in presence of over 38 mg/mL concentrations. Mohammed [36] evaluated the effect of ethanolic and ether extracts of N. sativa on S. mutans and Streptococcus mitis, and reported superior results for the ethanolic extract with a larger growth inhibition zone against S. mutans. However, it had lower effects on Streptococcus mitis; nonetheless, its ethanolic extract was more effective than its ether extract [36]. Dhahir Mansour Al Sultaniet al. [37] evaluated the MIC of the lipid and aqueous extracts of N. sativa on Gram-positive and Gram-negative bacteria and found their comparable antibacterial activity with no significant difference. Gram-negative bacteria were affected by double the concentration of extract compared with Gram-positive bacteria, indicating higher sensitivity of Gram-positive bacteria to the extract. In the present study, promising results were obtained regarding the antibacterial activity of the extract such that its MIC was 250 mg/mL for 1% and 2% concentrations and 125 mg/mL for 3%, 4%, and 5% concentrations. Also, its 5% concentration showed superior results to chlorhexidine control group in agar disc diffusion test. These results supported the findings of previous studies regarding optimal antibacterial activity of N. sativa [35][36][37]. Azimi Laysar et al. [38] showed optimal antibacterial effects of different concentrations of N. sativa extract and nano-silver on S. mutans and Streptococcus sanguinis. The growth inhibition zone caused by nano-silver was larger than that caused by N. sativa; nonetheless, the growth inhibition zone caused by N. sativa was still larger than that caused by amoxicillin.

In the present study, N. sativa honey had no significant inhibitory effect on S. mutans, and did not cause its proliferation (due to its sugar content) either. However, this result was in contrast to the findings of previous studies. Rezvani et al. [16] confirmed the synergistic effect of honey and cinnamon against S. mutans, and reported that this effect was not concentration-dependent. These inconsistent results can be caused by the difference in the composition of cinnamon with N. sativa or the difference in the synergistic effect of honey with each of the plant extract. The Nassar et al. [39] evaluated the effect of honey on S. mutans and biofilm formation. They evaluated both natural and synthetic honey, and showed that bacterial biofilm was easily detached from the wells that contained natural honey while the biofilm firmly adhered to the walls in wells containing synthetic honey. Ahmadi-Motamayelet al. [40] assessed the in vitro effect of honey in different concentrations on S. mutans and showed that honey in concentrations >20% had an inhibitory effect on S. mutans. It also inhibited Lactobacillus in 100% concentration. Deglovic et al. [41] demonstrated that honey prevented bacterial adhesion to tooth surface and could be even more effective than CHX. The antibacterial agents in the composition of honey include hydrogen peroxide, which is effective on both Gram-positive and Gram-negative bacteria, gluconic acid, which is effective against Gram-negative bacteria, and defensin-1 which is effective against both Gram-positive and Gram-negative bacteria [41].

Patelet al. [28] demonstrated that honey in concentrations <60% could not inhibit bacterial growth but had optimal antibacterial efficacy in higher concentrations. The growth inhibition zone diameter increased by an increase in its concentration. Roselena et al. [42] indicated an increase in antibacterial activity of honey against S. mutans by an increase in its concentration. Libonatti et al. [43] discussed that honeys obtained from different geographical areas had variable effects on the bacteria. For example, Iranian honey was effective on Staphylococcus aureus, and Pseudomonas aeruginosa while Malaysian honey was effective on Salmonella typhi and Shigellasonnei. Voidarou et al. [44] evaluated the effects of honeys obtained from different herbs on different bacteria, and showed that thyme honey was effective on Staphylococcus aureus with a growth inhibition zone of 4 mm and on Escherichiacoli with a growth inhibition zone of 6 mm; whereas, multi-herb honey had no significant effect on Staphylococcus aureus, and caused a 3 mm growth inhibition zone in Escherichia coli culture. Variations in the results of studies can be due to different origins of honeys.

This study had an in vitro design. Thus, the results can not be directly generalized to the clinical setting. Future studies are required to assess the effects of higher concentrations of N. sativa extract and find its safest and most effective concentration for possible incorporation in oral hygiene products. Also, the effects of N. sativa extract and honey on expression of colonization and biofilm formation genes of S. mutans should be investigated.

## Conclusion

N. sativa honey alone did not demonstrate any significant effect on inhibiting the growth of S. mutans. The combination of N. sativa and honey had an inhibitory effect on the growth of S. mutans. Increased concentration of N. sativa result in a greater inhibition of S. mutans growth.

## Conflict of Interest

The authors declare that they have no conflicts of interest related to this article.
